# Phylogeography of *Pterocarya hupehensis* reveals the evolutionary patterns of a Cenozoic relict tree around the Sichuan Basin

**DOI:** 10.48130/forres-0024-0005

**Published:** 2024-03-12

**Authors:** Zi-Jia Lu, Tian-Rui Wang, Si-Si Zheng, Hong-Hu Meng, Jian-Guo Cao, Yi-Gang Song, Gregor Kozlowski

**Affiliations:** 1 Eastern China Conservation Centre for Wild Endangered Plant Resources, Shanghai Chenshan Botanical Garden, Shanghai 201602, China; 2 College of Life Sciences, Shanghai Normal University, Shanghai 200234, China; 3 Plant Phylogenetics and Conservation Group, Center for Integrative Conservation, Xishuangbanna Tropical Botanical Garden, Chinese Academy of Sciences, Kunming 650223, China; 4 Southeast Asia Biodiversity Research Institute, Chinese Academy of Sciences, Naypyidaw 05282, Myanmar; 5 College of Forestry and Biotechnology, Zhejiang A&F University, Hangzhou 311300, China; 6 Department of Biology and Botanic Garden, University of Fribourg, Fribourg 1700, Switzerland; 7 Natural History Museum Fribourg, Fribourg 1700, Switzerland

**Keywords:** Phylogeography, Sichuan Basin, *Pterocarya hupehensis*, Demographic history, Wind-pollinated, Cenozoic relict species

## Abstract

Environmental factors such as mountain tectonic movements and monsoons can enhance genetic differentiation by hindering inter- and intra-specific gene flow. However, the phylogeographic breaks detected within species may differ depending on the different molecular markers used, and biological traits may be a major confounding factor. *Pterocarya hupehensis* is a vulnerable relict species distributed throughout the Sichuan Basin. Here, we investigated the phylogeographic patterns and evolutionary history of *P. hupehensis* using chloroplast DNA and restriction site-associated DNA sequencing data from 18 populations around the Sichuan Basin. The 24 chloroplast haplotypes separated into western and eastern lineages at approximately 16.7 Mya, largely coincident with a strengthening of the East Asian monsoon system during the early to middle Miocene. Both cpDNA and nuclear DNA datasets consistently identified distinct western and eastern lineages whose phylogeographic break conformed to the boundary of the Sino-Himalayan and Sino-Japanese forest sub-kingdoms. However, in contrast to the nuclear gene data, the cpDNA data revealed further divergence of the eastern lineage into northern and southern groups along the Yangtze River, a result that likely reflects differences in the extent of pollen vs seed dispersal. During the temperature decline in the penultimate (Riss) glacial period of the Pleistocene epoch, *P. hupehensis* experienced a genetic bottleneck event, and ecological niche modeling suggests that a subsequent population expansion occurred during the last interglacial period. Our findings not only establish a basis for conservation of this species, but also serve as a case study for the effects of geography and climate change on the evolutionary history of wind-pollinated relict plants.

## Introduction

The distribution and genetic structure of many species have been influenced by environmental factors such as monsoons, mountain tectonics, and other historical/ecological processes^[1−3]^. In addition, the presence of geographic barriers, combined with species-specific characteristics such as dispersal mode of seeds or pollen, can potentially effect the genetic differentiation of species^[4−6]^. Thus, understanding phylogeographic patterns and their potential influences on biomes is a primary objective of conservation and evolutionary biology^[1,7]^.

The Sino-Japanese Floristic Region (SJFR), known for its abundant diversity of temperate flora, has attracted significant attention from phylogeographers and paleo-ecologists^[8−10]^. Its high biodiversity is typically explained by the absence of continental glaciation and a smaller magnitude of Quaternary environmental change^[11]^. The Sichuan Basin, a unique geological structure in East Asia, acts as a mountain refuge for numerous relict species^[12−14]^. The phylogeographic break of relict plants around the Sichuan Basin is mainly related to uplift of the Qinghai-Tibet Plateau (QTP) during the late Pliocene, as well as intensification of the East Asian monsoon system (EAMS)^[15−17]^. Climate fluctuations since the Miocene may also have been a key determinant of the differentiation and colonization of ancient taxa^[18,19]^. However, the dynamics of species around the Sichuan Basin during the Miocene climate change—and how species characteristics and geographic barriers affected their genetic patterns—remain poorly understood.

The climate of Asia has changed dramatically since the Miocene^[20−22]^. Between the early and middle Miocene, the pattern of aridity began to change from 'planetary' subtropical to 'inland'^[21]^, and this was followed by formation and dominance of the monsoon climate^[23,24]^. This phenomenon is generally explained by uplift of the QTP^[25,26]^ and cooling of the global climate during the middle Miocene^[27]^, which further enhanced East Asian summer and winter monsoons during this period (*c*. 15–10 Mya)^[28−30]^.

Many phylogeographic studies have shown that several post-Miocene uplift and monsoon events are related to the genetic structure and genetic differentiation of plants^[14,18,31]^. Monsoons can also strengthen geographic barriers by giving rise to different climatic environments on either side of the barrier, promoting the formation of lineage discontinuities, such as the 'Tanaka–Kaiyong Line' (TKL)^[32,33]^. Abundant summer precipitation, brought about by strengthening of the East Asian monsoon, is critical to the development and maintenance of subtropical evergreen broad-leaved forests in China^[34,35]^. Aridity is also an important factor affecting plant distribution^[36]^. With uplift of the QTP and strengthening of the Asian monsoon in the Early Miocene, inland drought began to occur and intensify in Asia^[31,37,38]^. This forced plants in the interior of Asia to retreat southward to find suitable habitats in subtropical regions with better hydrothermal conditions.

The Sichuan Basin, which is located in the second step of China's terrain, is surrounded by mountains. These mountains exhibit complex geomorphological and climatic characteristics that have created diverse habitats for relict species along steep ecological gradients^[39,40]^. Many physical and ecological barriers, such as mountains, rivers, and climate, are believed to drive population diversification and speciation around the Sichuan Basin^[6,16]^. Among the main phylogeographic patterns around the Sichuan Basin, the best-known is the 105°E line^[6,41]^. This line runs longitudinally across the Sichuan Basin, coincident with the boundary of the Sino-Himalayan and Sino-Japanese forest sub-kingdoms, dividing taxa into eastern and western lineages^[13,42]^. The TKL is another major phytogeographic boundary that traverses the mountains in the southwestern Sichuan Basin^[43]^. In addition, the Yangtze River (Three Gorges region) traverses the mountains in the eastern Sichuan Basin, forming a phylogeographic break that divides taxa into northern and southern lineages^[31,44]^. Monsoons, QTP uplift, mountains, and river basins have thus isolated biodiversity into areas of endemism or created lineages by impeding gene flow in dispersal-limited organisms^[16,40,45]^.

Most phylogeographic studies of plants in East Asia have used chloroplast molecular markers^[14,17,18,46,47]^. Chloroplast DNA (cpDNA) is passed down by uniparental maternal inheritance in most angiosperms and is transmitted by seeds alone, thus providing no pollen-related information^[48]^. However, pollen-mediated gene flow may dominate in wind-pollinated trees^[5,49]^. Asymmetrical gene flows mediated by pollen and seeds have been consistently demonstrated in phylogenetic studies of many temperate tree species^[5,41,50]^. Different transmission characteristics may result in different intraspecific genetic structures. For example, inconsistencies between cpDNA and nuclear DNA of *Juglans cathayensis* and *Populus lasiocarpa* have been attributed to biological traits (e.g. extensive pollen exchange and wind-dispersed seeds) that partly delay the genetic imprinting of long-term isolation^[5,6]^. When exploring the effects of past events on the evolutionary history of taxa, species-specific biological characteristics should therefore be taken into account.

*Pterocarya hupehensis* is a tree species endemic to China that is found in mountainous areas (between 700 and 2,000 m above sea level) around the Sichuan Basin^[45,51,52]^. It is a typical riparian relict species of subtropical evergreen broad-leaved forests^[51]^. *P. hupehensis* is monoecious, producing flowers in catkins (amenta), with female amenta terminals on new growth; it is thus a typical anemophilic, cross-pollinated species^[53,54]^. *P. hupehensis* has been assessed as vulnerable (VU) and is considered to be of conservation concern^[52]^; a better understanding of its population genetics and phylogeography can therefore guide appropriate protective actions. It is also an excellent case study for investigating potential drivers of genetic patterns in wind-pollinated relict species around the Sichuan Basin.

Here, we used cpDNA and restriction site-associated DNA sequencing (RAD-seq) datasets to reconstruct the evolutionary history and phylogeography of *P. hupehensis*. We then performed ecological niche modeling (ENM) to explore its suitable habitats from the past to the future. In particular, we addressed three questions. First, what is the genetic structure of *P. hupehensis* populations around the Sichuan Basin? Second, how did this species respond to climatic fluctuations from the Neogene to the Quaternary periods? Third, what evolutionary processes contributed to the observed genetic patterns?

## Materials and methods

### Sampling and sequencing

Leaves were collected from 18 natural populations covering the distribution range of *P. hupehensis* around the Sichuan Basin (Table 1). Individuals in each population were at least 50 m away from each other, and 156 individuals were sampled. Fresh leaves were dried using silica gel, and DNA was extracted from the dried leaf tissue using a modified CTAB method^[55]^. After removal of samples with low-quality DNA, 141 individuals were selected for cpDNA sequencing and 122 individuals for RAD sequencing (Supplemental Table S1). Voucher specimens of each individual were stored at the Shanghai Chenshan Botanical Garden Herbarium (CSH). After screening previously published universal primers, six cpDNA loci (*psb*D-*trn*T^[56]^, *trn*V(UAC)x2-*ndh*C^[57]^, *trn*L(UAG)-*rpL*32-F^[56]^, *trn*S-*trn*fM^[58]^, *trn*G-*trn*S^[59]^, and *trn*D-*trn*Y^[60]^) were selected for use in this study. PCR amplification was performed as described previously^[61]^. The amplified PCR products were sequenced by Sangon Bioengineering Co., Ltd. (Shanghai, China).

**Table 1:** Sample codes, sample locations, and genetic diversity indices for 18 populations of *Pterocarya hupehensis* based on RAD-seq data and cpDNA data.

Code	Site	Longitude (°E)	Latitude (°N)	RAD-SNPs						cpDNA		
				*n*	*H*_O_	*H*_E_	π	*F*_is_		*h*	*π* × 10^3^	Haplotype (number of individuals)
HH	Xian, Shaanxi	107.93	33.87	6	0.199	0.173	0.203	0.007		0	0	H1 (10)
HXC	Ankang, Shaanxi	109.49	32.72	3	0.207	0.165	0.209	0.004		0.42	0.005	H2 (1), H3 (1), H4 (7)
FLC	Ankang, Shaanxi	109.4	31.95	5	0.192	0.143	0.174	−0.033		0	0	H5 (9)
DSP	Baoji, Shaanxi	107.49	33.84	4	0.183	0.144	0.177	−0.013		0	0	H6 (8)
QDZ	Nanyang, Henan	111.97	33.52	6	0.210	0.175	0.197	−0.027		0.39	0.004	H7 (7), H8 (2)
HKC	Nanyang, Henan	112.02	33.57	6	0.193	0.184	0.209	0.033		0.5	0	H7 (6), H9 (3)
TSG	Nanyang, Henan	111.72	33.63	6	0.182	0.171	0.198	0.031		0.33	0	H7 (5), H10 (1)
LJL	Nanyang, Henan	111.70	33.63	4	0.191	0.164	0.207	0.027		0	0	H7 (4)
LYG	Tianshui, Gansu	106.1	34.23	5	0.189	0.152	0.177	0.021		0	0	H1 (10)
YPC	Longnan, Gansu	106.23	33.67	5	0.206	0.149	0.170	0.068		0	0	H11 (7)
HJG	Kangxian, Gansu	105.51	33.39	5	0.184	0.141	0.162	0.041		0	0	H12 (7)
MYG	Yangba, Gansu	105.74	33.03	6	0.191	0.170	0.196	0.010		0	0	H13 (6)
SNJ	Shennongjia, Hubei	110.92	31.65	7	0.216	0.199	0.217	0.004		0	0	H14 (7)
XJZ	Shennongjia, Hubei	110.58	31.59	11	0.209	0.209	0.220	0.033		0.47	0.067	H15 (7), H16 (3)
SHJZ	Enshi, Hubei	109.8	30.16	11	0.206	0.179	0.194	−0.026		0.76	0.067	H17 (5), H18 (1), H19 (2), H20 (1), H21 (1)
JSZ	Nanchuan, Chongqing	107.14	29.02	7	0.213	0.212	0.223	0.029		—		—
DFX	Bijie, Guizhou	105.88	27.33	12	0.224	0.212	0.222	−0.001		0.36	0.004	H22 (8), H23 (2)
NYX	Bijie, Guizhou	105.47	26.7	13	0.227	0.195	0.204	−0.047		0	0	H24 (10)
AVERAGE					0.201	0.174	0.198	−0.006		0.19	0.009	
*n*, the number of samples used for RAD-seq analysis; *H*_O_, observed heterozygosity; *H*_E_, expected heterozygosity; π, nucleotide diversity; *F*_is_, inbreeding coefficient; *h*, haplotype diversity.												

### Chloroplast DNA sequence analysis

Chloroplast DNA sequences were assembled and checked using Sequencher v4.1.4 (Gene Codes Corp., Ann Arbor, MI, USA). The sequences were aligned and calibrated using ClustalW implemented in MEGA v11^[62]^ and then manually calibrated and adjusted. The haplotypes of the chloroplast fragments were extracted using DnaSP v6.0 with default parameters^[63]^, and a haplotype distribution map was constructed using ArcGIS v10.2. Haplotype diversity (*H*_d_) and nucleotide diversity (π) were calculated with Arlequin v3.5^[64]^. Total gene diversity (*H*_T_), within-population gene diversity (*H*s), and population differentiation indices (*G*_ST_ and *N*_ST_) were calculated using PERMUT v2.0^[65]^. The Median Joining model of NETWORK v10.2.0.0^[66]^ was used to construct the haplotype network. BARRIER v2.2 was used to detect biogeographic boundaries evaluated by 100 replicates of population average pairwise difference matrices^[67]^. Analysis of molecular variance (AMOVA) with 1,000 permutations was performed to examine genetic variation among and within populations using Arlequin v3.5^[64]^.

BEAST v2.5 was used to estimate chloroplast haplotype divergence times under a log-normal relaxed clock^[68]^. We chose *Juglans regia* as an outgroup^[69]^. On the basis of the Akaike information criterion (AIC) implemented in Modeltest v3.7^[70]^, the HKY + I + G model was selected as the best alternative model. The age of the earliest conclusive *Juglans* L. fossil was used as the minimum age to constrain the stem of the haplotype tree (~50 Mya: 44.4–57.88 Mya)^[71]^. We also used *Pterocarya fraxinifolia* and *Pterocarya macroptera* as outgroups, and the age of the earliest *Pterocarya* fossil was used to constrain the crown group (~30 Mya: 28–34 Mya)^[51]^. Markov chain Monte Carlo runs were performed for 10 million iterations with parameter sampling every 1,000 generations. Convergence was assessed using Tracer v1.7^[72]^, and the effective sample size (ESS) for all parameters was calculated. The first 20% of each run was discarded as burn-in using TreeAnnotator v1.8 (http://beast.bio.ed.ac.uk/TreeAnnotator). Mismatch distribution analysis and two neutrality tests, Tajima's *D*^[73]^ and Fu's *F*s^[74]^, were performed to estimate historical demographic expansions using Arlequin v3.5^[64]^.

### RAD-seq data processing and analysis

#### SNP calling and filtering

The raw reads from all 122 individuals were cleaned to remove reads with uncalled bases and low quality scores using the process_radtags module in Stacks v2.62^[75]^. The process_radtags module was also used to truncate the final reads to 120 bp. The *Pterocarya stenoptera*^[76]^ reference genome was indexed with BWA v0.7.17^[77]^, and the *P. hupehensis* RAD sequences were aligned to the indexed reference genome. The 'flagstat' command in SAMtools v1.16 was used to calculate the mapping rates and read numbers^[78]^. The Stacks v2.62 pipeline was used to process the RAD-seq reads^[75]^. The gstacks module was used to identify single nucleotide polymorphisms (SNPs) at each locus in the population and genotype each individual for each identified SNP. The resulting BAM files were sorted with SAMtools^[78]^. The populations module in Stacks was used for data filtering and SNP calling with the following criteria: (1) greater than 80% of individuals in each population were processed for each locus using the parameter 'r = 0.8'; (2) the maximum observed heterozygosity was set to 0.7 with the parameter 'max-obs-het = 0.7'; (3) the minimum minor allele frequency (MAF) was set to 0.05; and (4) only the first SNP locus of each read was retained to avoid physical linkage. The variant dataset was then filtered for missing data using VCFtools v0.1.16^[79]^ with the parameter 'max-missing = 0.8'.

#### Population structure and genetic diversity

Bayesian clustering was performed using Admixture^[80]^. The most probable values of *K* for explaining population structure were determined using the lowest cross-validation (CV) error rate. R v4.1.0^[81]^ was used to visualize the curve of the CV error rate from one to ten and the population structure histogram. Population structure was also investigated using principal component analysis (PCA) for 122 individuals with the R package 'adegenet'^[82]^. The optimal number of lineages was selected on the basis of the lowest associated Bayesian information criterion. An individual-based maximum likelihood (ML) tree was constructed using IQ-TREE v1.6.12^[83]^ and contained three outgroups: *P. macroptera*, *Cyclocarya paliurus*, and *Juglans mandshurica*^[45]^. The nucleotide diversity (π), the expected and observed heterozygosities (*H*_E_ and *H*_O_), and the fixation index (*F*_is_) among populations were calculated using the populations module in Stacks. AMOVA was performed using Arlequin v3.5^[64]^ to explore the degree of genetic differentiation among lineages, populations within lineages, and populations.

#### Population demographic histories

We used TreeMix v1.13^[84]^ to infer possible hybridization events among populations by obtaining allele frequencies from multiple populations and generating a ML tree. Migration events were analyzed from one to ten and then calibrated on the ML tree. The parameter '-noss' was used to prevent overcorrection. We calculated the percent variance explained in order to judge the migration events using the script 'treemixVarianceExplained.R'. We set the program to use 10 migration events for generation of the ML tree. The standard errors of all entries in the covariance matrix estimated from the data were used to construct a heatmap. The migration tree and heat map were visualized using R v4.1.0.

Fluctuations in effective population size were inferred using Stairway Plot 2^[85]^, which implements an unsupervised learning strategy for model selection and supports both folded and unfolded site frequency spectra (SFS). The *P. stenoptera* reference genome was indexed, and unfolded SFSs were generated for both genetic groups and total populations using ANGSD v0.939^[86]^. The effective population size was inferred for each lineage and for all populations using a mutation rate of 2.06 × 10^−9^ per locus per year (with reference to *Juglans*)^[87]^ and a generation time of 30 years. We used the recommended percentage of training sites (67%) to run the Stairway Plot 2 program. By default, 200 input files were created for each estimation.

BEAST v2.5^[68]^ was used to estimate the nuclear genome (RAD-seq) divergence time for *P. hupehensis*. According to the phylogeny of Juglandaceae, *Cyclocarya paliurus* and *Pterocarya macroptera* were used as outgroups^[71]^. We used the oldest fossil of *Cyclocarya* to calibrate the stem age of *Pterocarya* (65–55 Mya)^[51]^ and the oldest fossil of *Pterocarya* to calibrate the crown age of *Pterocarya* (34–28 Mya)^[51]^. MCMC chains were run for 50,000,000 generations under the GTR + I + G model chosen by jModelTest^[88]^. Tracer v1.7 was used to evaluate convergence and calculate the ESS (http://tree.bio.ed.ac.uk/software/tracer/). The first 30% of samples were discarded as burn-in using TreeAnnotator v1.8, and the phylogeny was visualized using Figtree v1.4.4 (http://tree.bio.ed.ac.uk/software/Figtree/).

### Ecological niche modeling

The potential distribution areas of *P. hupehensis* were generated on the basis of all known distribution points using M_AXENT_ v3.4.4^[89]^. The distribution points included the 18 sampling points used here, as well as distribution records from the National Specimen Information Infrastructure (www.nsii.org.cn/) and the Chinese Virtual Herbarium (www.cvh.ac.cn). We deleted points corresponding to non-natural populations (parks, urban areas, etc.) using a coordinate backcheck. The R package 'dismo'^[90]^ was used to delete missing, duplicate, and incorrect coordinates. To reduce the influence of spatial autocorrelation on climate variables, we performed grid screening to remove coordinate points less than 2.5 arc-min (~4.6 km) apart using the R package 'raster'^[91]^. We acquired the standard 19 bioclimatic variables from the WorldClim website (https://worldclim.org/) at a 2.5-arc-min spatial resolution^[92]^ for four time periods: the present (1970–2000), the last glacial maximum (LGM, *c*. 21 kya)^[93]^, the last interglacial period (LIG, *c*. 120 kya)^[94]^, and the 2061–2080 period under the representative concentration pathway (RCP) 4.5 scenario. We used the function variance inflation factor (VIF) from the R package 'usdm'^[95]^ to remove environmental factors with correlation coefficients > 0.8. The following environmental factors were retained as predictors: annual mean temperature, mean diurnal range, temperature seasonality, precipitation of wettest month, precipitation seasonality, and precipitation of driest quarter. In total, 25% of the data were used for model testing and validation. Ten independent replicates were analyzed using the bootstrap method. The average results were used to reclassify the suitable areas in ArcGIS v10.2. The parameter for reclassification had five levels, 0–0.08, 0.08–0.25, 0.25–0.5, 0.5–0.75, and 0.75–1, indicating the degree of habitat suitability from low to high.

## Results

### cpDNA sequence variation

**Figure 1:**
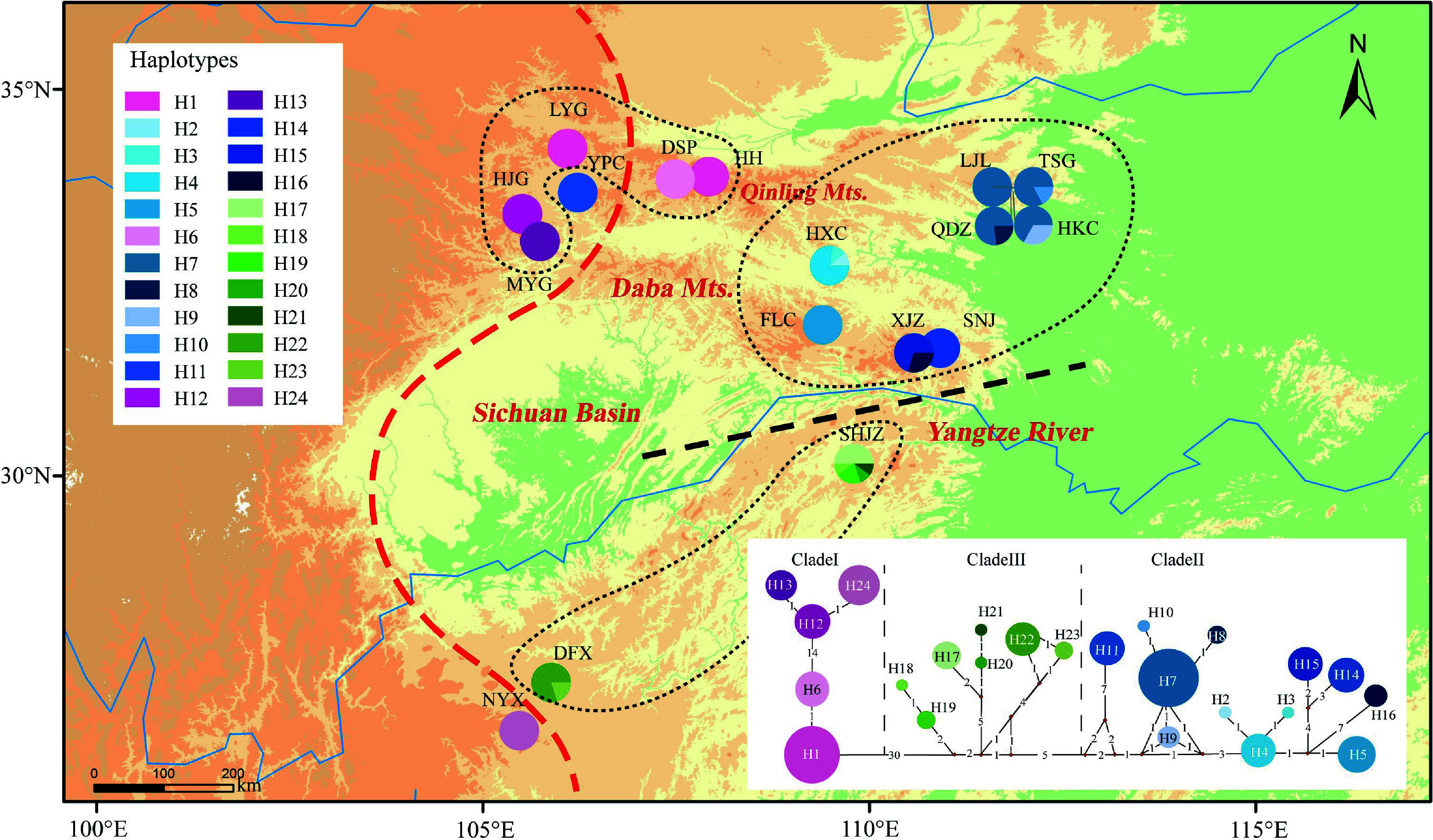
Distribution of 24 chloroplast haplotypes of *Pterocarya hupehensis*. The lower right panel shows the minimum spanning network of 24 chlorotypes. Circle sizes are proportional to the number of samples per haplotype. The red dashed line on the map represents the Sino-Himalayan/Sino-Japanese forest boundary. The black dashed line represents the phylogeographic break along the Yangtze River in the eastern Sichuan Basin. The black dotted lines delineate three phylogroups with closely related chlorotypes.

The alignments of the *psb*D-*trn*T, *trn*V-*ndh*C, *trn*L-*rpL*32, *trn*S-*trn*fM, *trn*G-*trn*S, and *trn*D-*trn*Y sequences were 1,578, 709, 849, 1305, 1,524, and 780 bp in length, respectively. After concatenation, the resulting 6,745 bp of chloroplast DNA sequence was found to contain 91 polymorphisms and 24 haplotypes in the 17 examined populations (Table 1, Fig. 1). The haplotype diversity (*H*_d_) and nucleotide diversity (*π*) of *P. hupehensis* were 0.929 and 0.00379, respectively. Population SHJZ had the highest haplotype diversity (*h* = 0.76), and populations XJZ and SHJZ had the highest nucleotide diversity (π = 0.0067) (Table 1). Total diversity (*H*_T_ = 0.963) was much higher than the average within-population diversity (*H*_S_ = 0.189). Significant phylogeographic structure was detected among the populations (*G*_ST_ = 0.804 < *N*_ST_ = 0.969, *P* < 0.05). The BARRIER analysis revealed genetic barriers between the eastern and western lineages (near the Qinling Mountains and the southern 105°E line) and also between the northern and southern Yangtze River (Supplemental Fig. S1). AMOVA showed that 75.05% of the total genetic variation occurred between the two lineages (*F*_CT_ = 0.75), 22.64% occurred among populations within lineages (*F*_SC_ = 0.91), and only 2.31% occurred within populations (*F*_ST_ = 0.98; Table 2).

**Table 2:** Analyses of molecular variance (AMOVA) based on cpDNA data and RAD-seq data for *Pterocarya hupehensis* from western and eastern lineages.

Source of variation	cpDNA				RAD-SNPs		
	d.f.	Percentage variation (%)	Fixation indices		d.f.	Percentage variation (%)	Fixation indices
Among lineages	1	75.05	*F*_CT_ = 0.75		1	22.22	*F*_CT_ = 0.22
Among populations within lineages	15	22.64	*F*_SC_ = 0.91		16	11.47	*F*_SC_ = 0.15
Within populations	124	2.31	*F*_ST_ = 0.98		226	66.31	*F*_ST_ = 0.34

**Figure 2:**
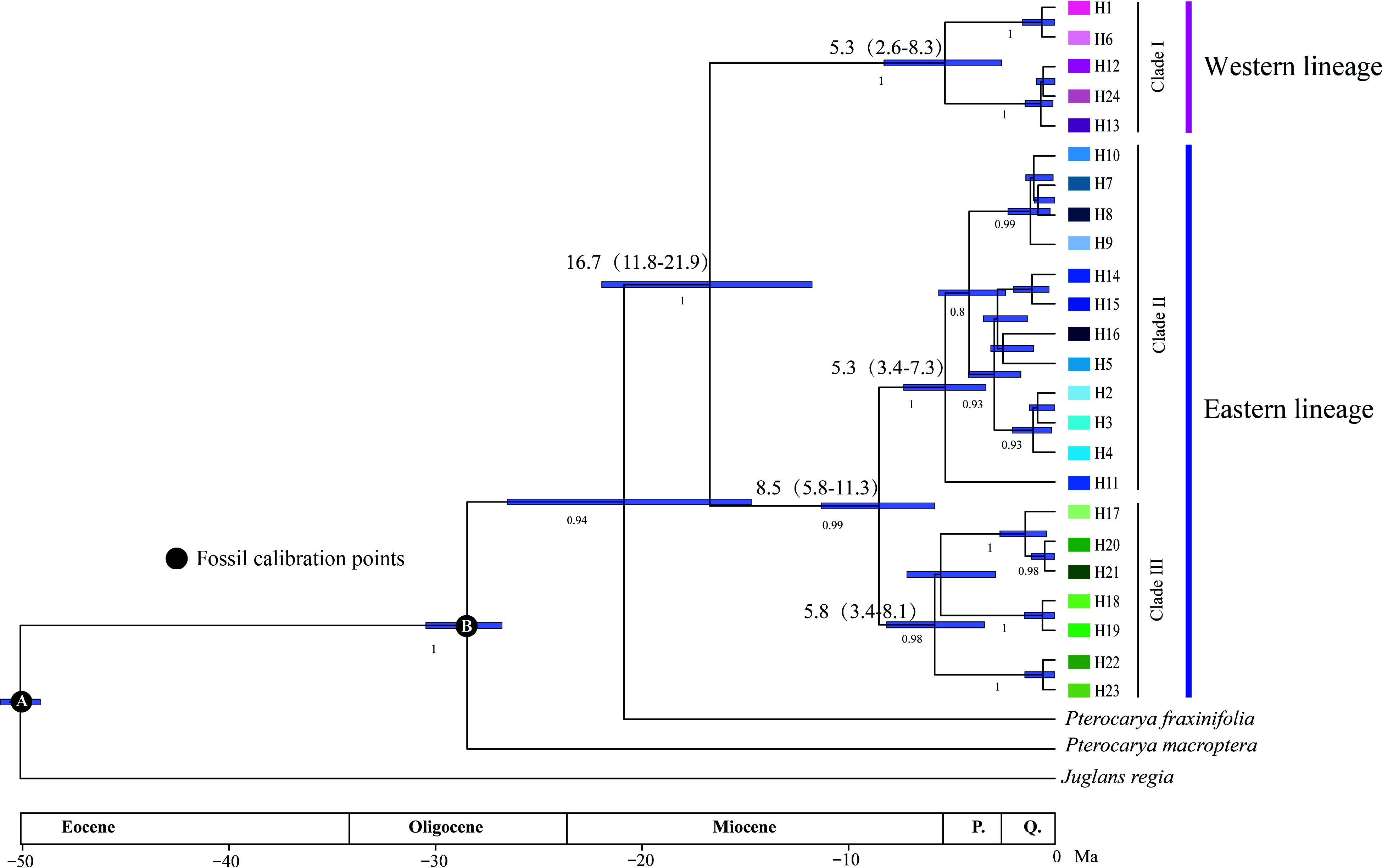
BEAST-derived chronogram of 24 *Pterocarya hupehensis* haplotypes based on six chloroplast DNA (cpDNA) fragments. The divergence time are shown above branches, with blue bars indicating the 95% highest posterior densities (HPDs). Posterior probabilities are labeled at each node.

The phylogenetic network resolved two main haplotype lineages with 30 step mutations (western and eastern lineages) located in the eastern and western parts of the Sichuan Basin. The eastern lineage was further divided into two clades (clades II and III). Clade II included haplotypes from the eastern Sichuan Basin and the western Qinling population (YPC). Clade III was composed mainly of haplotypes from the southeastern Sichuan Basin (Fig. 1). The haplotype structures revealed by BEAST were similar to the groupings revealed by the phylogenetic network. The haplotype divergence between the western and eastern lineages dated to the middle Miocene (16.7 Mya), and clade II separated from clade III at approximately 8.5 Mya. The crown age of clade II was estimated at 5.3 Mya and that of clade III at 5.8 Mya (Fig. 2).

The values of Tajima's *D* and Fu's *F*s were positive for *P. hupehensis* (*D* = 0.675, *P* = 0.693; *F*_S_ = 0.196, *P* = 0.626), suggesting no expansion of its distribution. Mismatch distribution analysis showed multimodal distributions for all samples, also suggesting that this species has not undergone a recent demographic expansion (Supplemental Fig. S2).

### Population structure and genetic diversity

We obtained 87 million RAD-seq reads from 122 samples. The average alignment rates of the samples to the reference genome was 86.72% (Supplemental Table S2). After restricting variants to those processed in > 80% of the individuals in each population, 695,262 variant sites remained. After filtering, 2,427 and 2,889 SNPs with and without outgroups, respectively, were obtained for subsequent analyses.

**Figure 3:**
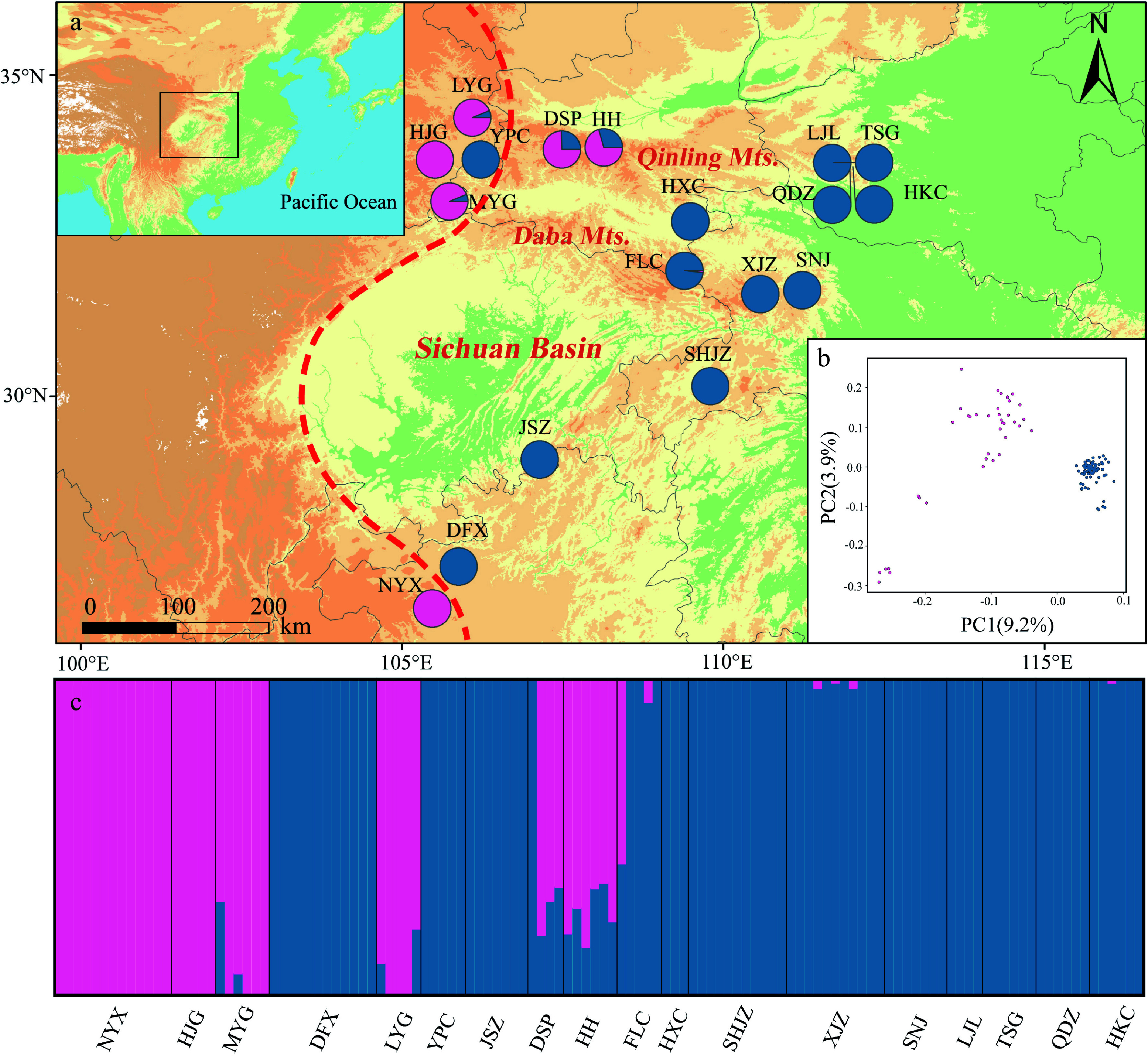
Genetic structure of *Pterocarya hupehensis* based on 2,889 SNPs dataset. (a) Geographic origins of 18 *P. hupehensis* populations and their color-coded grouping at close to *K* = 2. The red dashed line represents the Sino-Himalayan/Sino-Japanese forest boundary. (b) Principal component analysis (PCA), with pink and blue colors representing two clusters. (c) Histogram of the Admixture analysis for *P. hupehensis* with *K* = 2.

Admixture analysis revealed that the genetic structure of *P. hupehensis* consisted of two lineages (Fig. 3; Supplemental Fig. S3). Populations located in the northwestern Qinling Mountains, together with one population (NYX) from the southwest, were assigned to the western lineage. The eastern lineage included all populations from the eastern Sichuan Basin, as well as one population from the northwest (YPC) (Fig. 3a). Genetic introgression was detected in five populations (western lineages LYG, MYG, DSP, and HH and eastern lineage FLC) (Fig. 3a, c). The core populations of the eastern lineage always clustered together when *K* = 2 to 5 (Supplemental Fig. S4). PCA also divided all populations into two lineages. The percentages of variation explained by PC1 and PC2 were 9.2% and 3.9%, respectively (Fig. 3). A ML tree showed that the eastern lineage evolved earlier than the western lineage (Supplemental Fig. S5), and the hybrid populations were primarily located at the branch ends of the ML tree.

Genetic diversity of nuclear DNA varied among populations as assessed by *H*_O_ (0.182–0.227), *H*_E_ (0.141–0.212), π (0.162–0.223), and *F*_is_ (−0.047–0.068) (Table 1). Genetic diversity was higher in the southern populations, including NYX, DFX, and JSZ. AMOVA revealed that genetic differentiation occurred mainly within populations (*F*_ST_ = 0.34, 66.31%; Table 2).

### Population demographic histories

**Figure 4:**
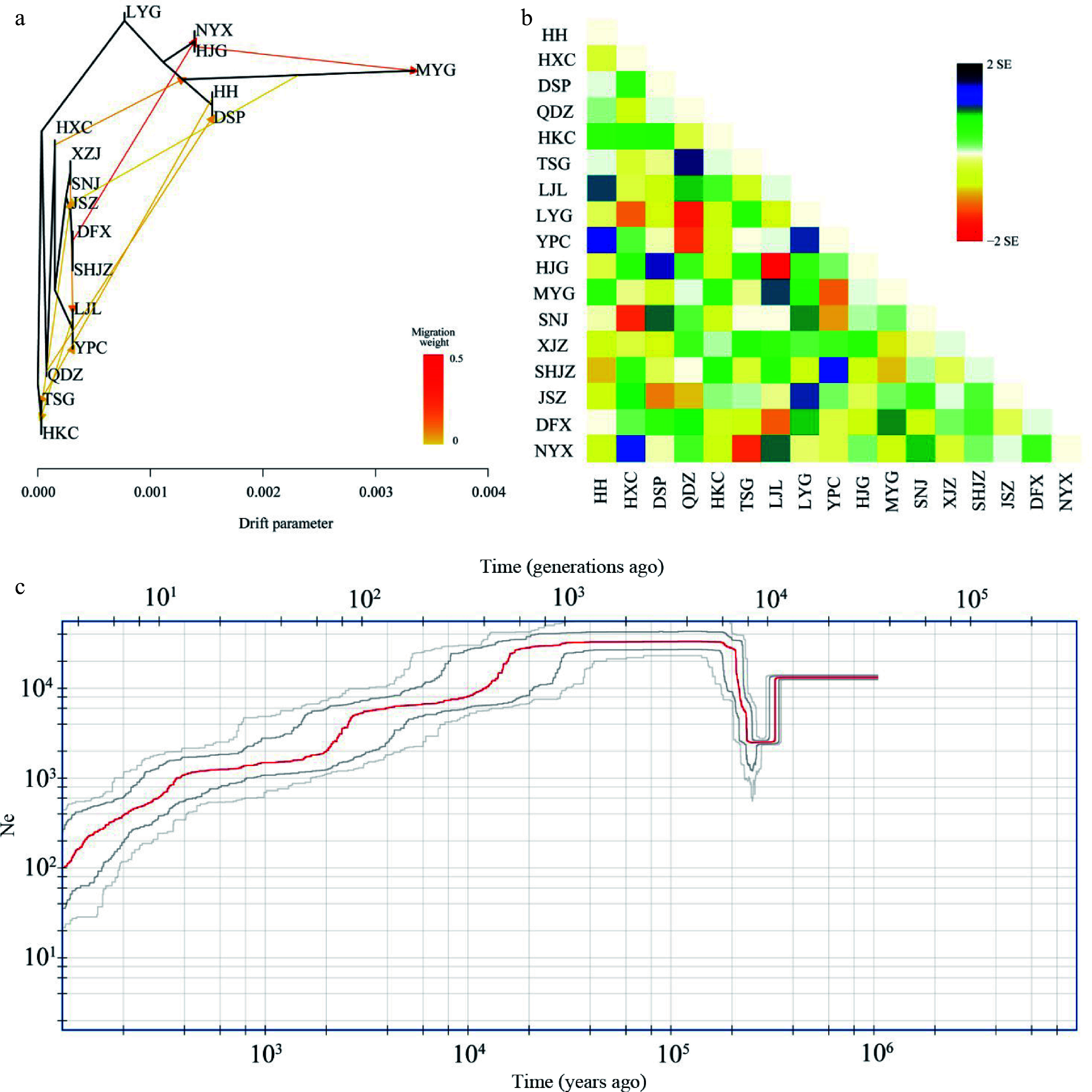
Hybridization among populations of *Pterocarya hupehensis*. (a) Maximum likelihood (ML) tree inferred using Treemix; gene-flow events are depicted with arrows, and ten migration events were allowed. Migration arrows are colored according to their weight. Horizontal branch lengths are proportional to the amount of genetic drift that occurred on each branch. The scale bar shows 10× the average standard error of the entries in the sample covariance matrix. (b) Heatmaps of residual fit from the ML tree. Residuals white through blue indicate that the corresponding populations are more closely related to each other than on the ML tree, suggesting confounding events between these populations. (c) Demographic history of *P. hupehensis* inferred with Stairway Plot 2 using unfolded site frequency spectra. The 95% confidence interval for estimated effective population size is shown with gray lines.

The migration events in the ML tree showed two strong signals with a high migration weight, indicating unidirectional gene flow from the DFX population to the common ancestral populations of NYX and HJG and also from the HJG to MYG populations (Fig. 4a). Eight other gene flow signals were also detected. The longest horizontal branch was that of the MYG population, indicating that it had undergone the greatest genetic drift compared with the other populations (Fig. 4a, Supplemental Table S3). The topology of the genetic relationships in the ML tree was consistent with the results of Admixture and IQ-TREE analyses. Stronger introgression events among populations were shown by the residual heatmap than by the ML tree inferred with Treemix (especially in the SHJZ, YPC, DFX, and QDZ populations) (Fig. 4a, b). The BEAST result based on the phylogeny from the RAD-seq datasets showed that the western lineage separated from the eastern lineage at 16.78 Mya (95% HPD: 11.99–22.25 Mya) (Supplemental Fig. S6), very similar to the divergence time of haplotypes between the western and eastern lineages estimated from cpDNA data (16.7 Mya, 95% HPD: 11.8–21.9 Mya).

Fluctuations in the effective population size of *P. hupehensis* were estimated to have occurred from approximately 1.0 Mya in the Late Pleistocene (Fig. 4c). Stairway Plot 2 analysis revealed a genetic bottleneck event at about 200–400 kya in which the effective population size dropped to about 1.4 × 10^4^ to 2 × 10^3^ individuals. The effective population size then climbed to an upper limit of ~3.2 × 10^4^ individuals at about 200 kya and remained steady between 120–140 kya during the LIG. From the LGM period to the Holocene, effective population size gradually decreased to its lowest level of 0.1 × 10^3^ individuals (Fig. 4c). The western and eastern lineages exhibited similar trends in population size fluctuation. However, the bottleneck event occurred slightly earlier in the western lineage than in the eastern lineage (Supplemental Fig. S7a, b).

### Ecological niche modeling

**Figure 5:**
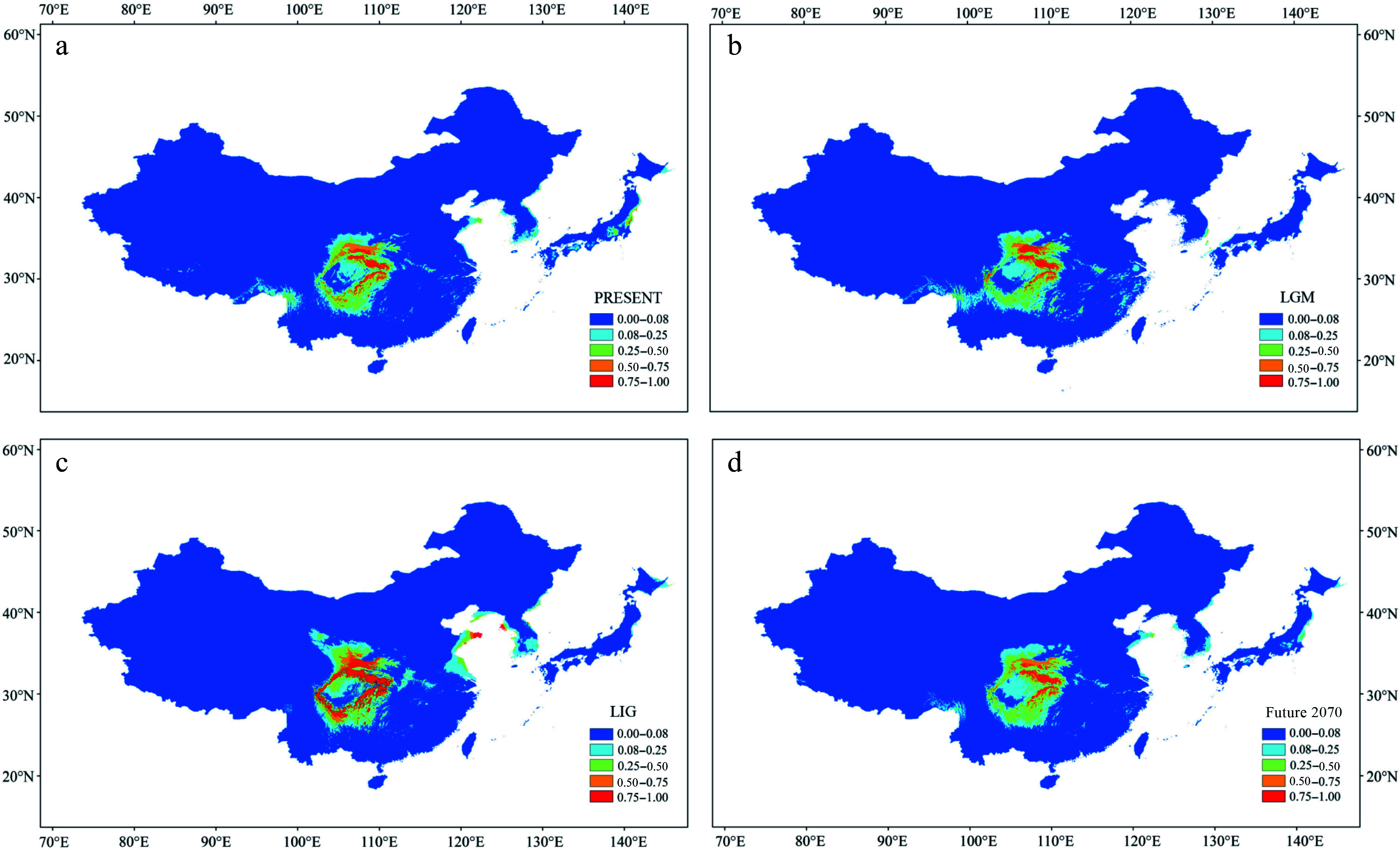
Results of ecological niche modeling of *P. hupehensis* in four time periods from past to future. (a) Average projection of the model to present climatic conditions. (b) Average projection of the model to the last glacial maximum (LGM: *c.* 21 kya BP (before present)). (c) Average projection of the model for the last interglacial (LIG: *c.* 120–140kya BP). (d) Average projection of the model to the year 2070 (2061–2080) under an intermediate climate warming scenario (RCP 4.5). Colors from blue to red represent the degree of habitat suitability for *P. hupehensis* survival, from unsuitable to suitable.

The Area Under Curve (AUC) value of the receiver operating characteristic (ROC) curve was high (> 0.977) in the four periods (Supplemental Fig. S8a–d). Annual mean temperature (35%) and mean diurnal range (26%) made the greatest contributions to the model under the current climate (Supplemental Fig. S9, Supplemental Table S4). The potential distribution of *P. hupehensis* under the present climate matched its current distribution; suitable area for its growth was limited to the mountains around the Sichuan Basin, especially in the northern and eastern regions (Fig. 5a). During the LGM period, the suitable area was significantly smaller in the southern and western regions of the Sichuan Basin but greater in the Qinling Mountains (Fig. 5b). The suitable area for *P. hupehensis* around the Sichuan Basin was greatest during the LIG period (Fig. 5c). Notably, during this period, an area of high suitability appeared in the Hengduan and Daliang Mountains. Under the RCP 4.5 scenario for 2061–2080, the suitable area for *P. hupehensis* was predicted to be just slightly smaller than at present, and the suitable areas in the western and southern Sichuan Basin were predicted to shrink under global warming (Fig. 5d).

## Discussion

### Phylogeographic conflicts between chloroplast and nuclear DNA

#### Significant chloroplast genetic structure

*Pterocarya hupehensis*, like other relict tree species in subtropical China, shows strong phylogeographic structure (*N*_ST_ > *G*_ST_) based on cpDNA markers^[5,41,42]^. Most of the haplotypes are private, except for H1 and H7, which are shared among populations. As reported for other angiosperm species, genetic differentiation among lineages of *P. hupehensis* was higher when assessed with cpDNA (*F*_CT_ = 0.75) than with nuclear DNA (*F*_CT_ = 0.22). Such significant chloroplast genetic structure is typically attributed to maternal inheritance, as chloroplasts are only transmitted by seeds, which have a limited dispersal distance. Seed-mediated gene flow can be influenced by seed dispersal ability, germination and dormancy, seedling establishment ability, and so forth. The wingnuts of *P. hupehensis* can be carried over short distances by wind and dispersed over long distances along rivers. *P. hupehensis*, as a relict species, is present in restricted microhabitats and shows strong niche conservatism (moist riparian forests along rivers with an elevational range of 700–2,000 m in the mountains)^[45,96−99]^. It produces relatively few seedlings, even when abundant seeds are present, owing to low seed quality and difficult seedling establishment^[51,52]^. Taken together, these factors have impeded the exchange of chloroplast genes among populations and shaped current genetic patterns.

#### Different nuclear genetic structure

Optimal clustering based on nuclear genetic structure (from RAD-seq data) divided *P. hupehensis* into two genetic clades in both Admixture analysis and PCA. More subdivided genetic structures were recognized in the Admixture analyses when *K* values were larger. Most populations in the eastern lineage were maintained in a single group from *K* = 2 to *K* = 5, indicating extensive gene flow *via* pollen dispersal. However, the numerous and private chloroplast haplotypes in the eastern lineage suggested their independent evolution (Fig. 1, Supplemental Fig. S3). Genetic differentiation among all populations was higher for cpDNA (*F*_ST_ = 0.98) than for nuclear DNA (*F*_ST_ = 0.34). Compared with maternal inheritance of cpDNA, parental inheritance of nuclear DNA typically exhibits more imprints from pollen flow. Thus, the inconsistency in genetic structure between cpDNA and nuclear DNA (especially for wind-pollinated species) can be understood as arising from differences between seed-mediated and pollen-mediated gene flows^[5,100,101]^. The present study provides another example of a temperate tree species with stronger genetic structure in the chloroplast genome (seed-mediated gene flow) than in the nuclear genome (pollen-mediated)^[42,102,103]^. Gene flows mediated by long-distance pollen dispersal were detected here and have been demonstrated in many other anemophilous tree species, such as *P. fraxinifolia*, *Quercus robur*, *Zelkova carpinifolia*, and others^[104−106]^. The strong East Asian monsoon that began in the early Miocene may have enabled the spread of pollen over long distances and thus promoted gene flow among populations^[21]^.

Extensive pollen flows of anemophilous tree species have been reported to facilitate genetic exchange and delay genetic differentiation in species with restricted distributions^[5]^. Such genetic exchange can improve population adaptation, particularly for tree species with slow evolutionary rates, high pollen dispersal capacity, and weak reproductive ability^[107,108]^. Compared with other wind-pollinated species, *P. hupehensis* has a relatively high level of genetic differentiation^[109,110]^, which may reflect the influence of slower pollen-mediated gene flow, higher levels of genetic drift, and local adaptation due to selection pressure associated with long-term environmental heterogeneity^[109−111]^. The bottleneck event and small population sizes of *P. hupehensis* may have led to high levels of genetic drift. In addition, long-established small and isolated populations of *P. hupehensis* are likely to have experienced more environmental selection pressure, which may also have contributed to a high level of genetic differentiation^[112,113]^.

### Species differentiation in the early to middle Miocene and later diversification

#### Colonization occurs during the wet and rainy monsoon

Reconstructions of divergence times based on cpDNA and nuclear DNA revealed that the eastern and western lineages of *P. hupehensis* diverged during the early to middle Miocene. A similar pattern was reported for *Cyclocarya paliurus*, which also belongs to the Juglandaceae^[18]^. By contrast, most relict tree species in this area diverged during the Pliocene, including *Davidia involucrata* (4.81 Mya), *Euptelea pleiosperma* (3.64 Mya), and *Populus lasiocarpa* (3.66 Mya)^[6,13,42]^. Initial intensifications of the East Asian summer and winter monsoons began in the early Miocene owing to the rapid uplift of the Tibetan Plateau^[20,28]^. Abundant precipitation associated with the monsoons, together with subsequent cooling during the mid-to-late Miocene and early Pliocene, promoted speciation and lineage differentiation of plants in East Asia^[16,114,115]^. The SJFR contains a rich diversity of temperate flora, which benefited from the changes in precipitation pattern and incomplete glacial coverage of the Quaternary glaciation^[10, 116−118]^. Our species distribution modeling provides further evidence that temperature and precipitation are the most important climatic predictors of suitable habitat for *P. hupehensis*. Both *P. hupehensis* and *C. paliurus* inhabit wet habitats near riverbanks or streams with high-humidity microclimates. Previous research has suggested that the characteristic of naked buds on temperate trees, as exhibited by *P. hupehensis*, may be associated with colder temperatures and summer precipitation^[18,119]^.

#### Multiple driving forces for lineage differentiation

The Sichuan Basin acts as a geographic barrier between the Sino-Himalayan and Sino-Japanese Forest subkingdoms (more or less along the 105°E line)^[120,121]^, affecting patterns of genetic diversity and structure for many relict species (e.g., *Davidia involucrata*^[13]^, *Dysosma versipellis*^[17]^, and *Primula ovalifolia*^[122]^). Both chloroplast and nuclear evidence demonstrate that *P. hupehensis* has also been influenced by this geographic barrier. The NYX and DFX populations provide a clear illustration of this barrier, as they are close geographically but contain chloroplast haplotypes and nuclear genes from different lineages. The western and eastern lineages of *P. hupehensis* appear to have diverged at the end of the early Miocene, which was followed by intensification of the EAMS. A previous study also detected a monsoon-driven phylogeographic break between western and eastern lineages of relict species around the Sichuan Basin^[16]^.

North–south lineage divergence in the Three Gorges region of the east Sichuan Basin has been documented in plants^[14,31,44]^ and animals^[121]^. Here, chloroplast haplotypes in the eastern lineage of *P. hupehensis* were further divided into northern and southern clades by the Yangtze River in the Three Gorges region. The barrier of the Three Gorges region blocks gene flow by limiting seed dispersal and animal migration. The absence of this phylogeographic break in nuclear gene analyses can be attributed to long-distance pollen dispersal by wind, and the results presented here for *P. hupehensis* are a good example of this phylogeographic inconformity.

The origin and formation of the Yangtze River and the Sichuan Basin were synchronized with the uplift of the Tibetan Plateau^[123]^, although details of the age and developmental history of the Yangtze River have been vigorously debated for more than 100 years^[124]^. Our results suggest that the Three Gorges barrier to plant gene flow may be traced back to the late Miocene, 5 Mya before the speculated formation time of the Yangtze River^[125]^. Thus, the phylogeographic break for some relict trees in the Three Gorges region may have occurred in the late Miocene. Overall, our data suggest that significant changes in climate and geography around the Sichuan Basin promoted phylogeographic breaks between the western and eastern lineages, whereafter in the eastern lineage of *P. hupehensis*.

#### Population demographic history after the Pleistocene

Climatic fluctuations in the Pleistocene glacial and interglacial periods had substantial effects on many relict plants. *P. hupehensis* experienced a population expansion during the warm LIG period and a slight shrinkage during the LGM period. However, we estimated relatively little fluctuation in its distribution around the Sichuan Basin from the Pleistocene to the future, consistent with findings for some other species^[6,121]^. Because the micro-environment of the mountains around the Sichuan Basin may have been more stable than that of other regions, East Asia acted as a biodiversity sanctuary during the LGM period. There was a brief cooling period at 120–350 kya (penultimate [Riss] glacial period) before the longest period of Pleistocene warmth^[126]^, and we detected a bottleneck event during this glacial period (200–400 kya). We speculate that temperature may have had an important effect on the distribution of *P. hupehensis*. The bottleneck effect can cause a series of negative chain reactions, especially for small populations, resulting in a loss of genetic diversity^[127,128]^, high levels of population isolation^[129]^, and altered fitness because of genetic drift and inbreeding^[130]^. The occurrence of bottlenecks or adversity is likely to lead to high levels of genetic drift^[127,131]^.

Our field investigations revealed that the current population of *P. hupehensis* is small and fragmented, with only a few dozen individuals in some populations^[52]^. *P. hupehensis* is currently listed as vulnerable on the IUCN Red List of Threatened Species. Our findings highlight the risk of a gradual decline in effective population size in the event of renewed adversity (Fig. 4c). Thus, future work should aim to assess the genomic vulnerability of each population, and both *ex situ* and *in situ* conservation of these small populations should be improved^[111]^. We should focus not only on the effect of global warming and greenhouse gas emissions on this species but also on the interference of human activities with its natural habitat.

## Conclusions

We used cpDNA and nuclear DNA data to reconstruct the phylogeographic history of *P. hupehensis.* Both cpDNA and nuclear genetic data revealed two distinct lineages corresponding to two phylogeographic regions. However, the cpDNA data suggest a relatively isolated and stronger phylogeographic structure than the nuclear data. This result suggests that pollen flow plays a more important role than seed flow in shaping genetic structure. External geologic and climatic changes have also influenced current genetic distribution patterns. Strengthening of the EAMS during the early to middle Miocene appears to have been the main driver of colonization and differentiation in *P. hupehensis*. The Three Gorges region, which acts as a seed dispersal barrier, promoted further north–south differentiation among the eastern lineages. In addition to population genetics studies and modeling, more efforts should be directed toward searching for *P. hupehensis* populations in the western Sichuan Basin. Genetic resources of *P. hupehensis* from the western and southwestern Sichuan Basin should be given priority.

## SUPPLEMENTARY DATA

Supplementary data to this article can be found online.

## Data Availability

The haplotype sequences of chloroplast DNA and the RAD-seq data have been deposited at the National Center for Biotechnology Information (NCBI) with GenBank accession numbers QQ884193–QQ884252 and PRJNA967132.
