# Supplementary material for: Phylogeography of Pterocarya hupehensis reveals the evolutionary patterns of a Cenozoic relict tree around the Sichuan Basin
Source: For Res (Fayettev). 2024 Mar 12;4:e008. doi: 10.48130/forres-0024-0005 (PMC11524273; doi:10.48130/forres-0024-0005)

Fig. S1 Map of genetic barriers to chloroplast haplotypes between different sampling areas. The blue lines show a Voronoï tessellation of populations according to geographic locations, and red solid lines represent genetic barriers. Circles represent the locations of the 18 *Pterocarya hupehensis* populations and are labeled with their population ID numbers.

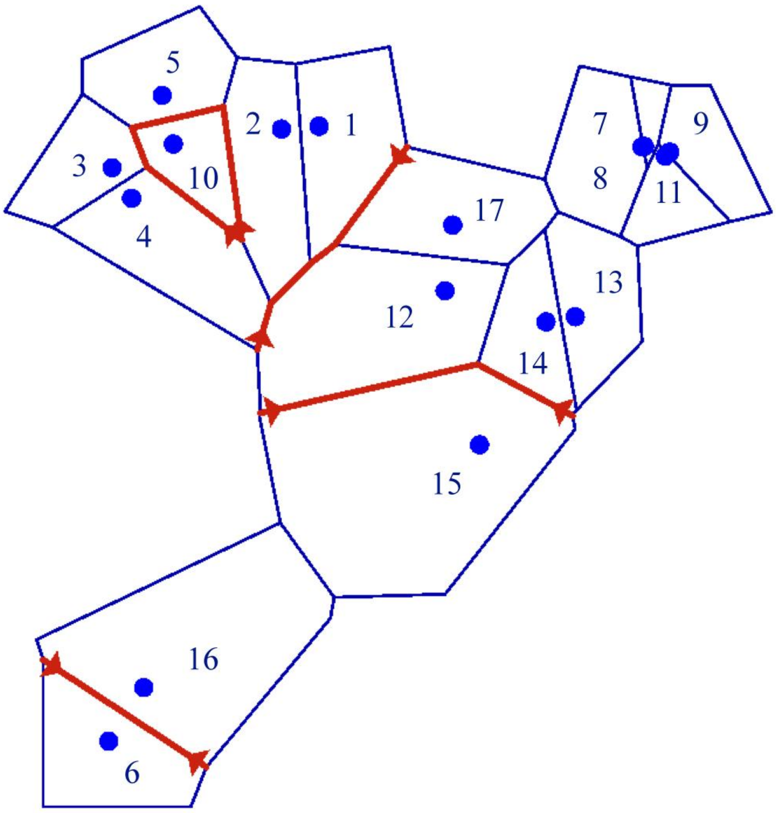

Supplement: Supplementary file 1 — Supplementary data to this article can be found online. [file forres-0024-0005-S1.zip › 10.48130_forres-0024-0005-Suppl-FigureS1.pdf]
