# Supplementary material for: Phylogeography of Pterocarya hupehensis reveals the evolutionary patterns of a Cenozoic relict tree around the Sichuan Basin
Source: For Res (Fayettev). 2024 Mar 12;4:e008. doi: 10.48130/forres-0024-0005 (PMC11524273; doi:10.48130/forres-0024-0005)

Fig. S2 Mismatch distribution for populations of *Pterocarya hupehensis*. The black and gray lines represent the observed and expected mismatch distributions, *r* represents the raggedness index, and *p* represents the significance of a simulation value greater than the observed value.

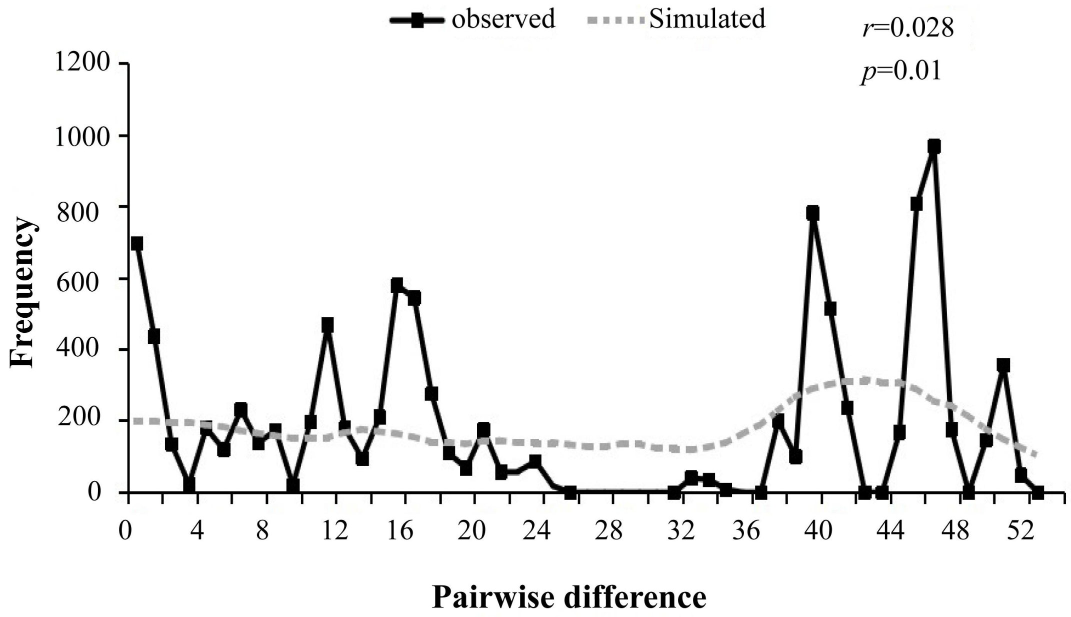

Supplement: Supplementary file 1 — Supplementary data to this article can be found online. [file forres-0024-0005-S1.zip › 10.48130_forres-0024-0005-Suppl-FigureS2.pdf]
