# Supplementary material for: Phylogeography of Pterocarya hupehensis reveals the evolutionary patterns of a Cenozoic relict tree around the Sichuan Basin
Source: For Res (Fayettev). 2024 Mar 12;4:e008. doi: 10.48130/forres-0024-0005 (PMC11524273; doi:10.48130/forres-0024-0005)

Fig. S4 Genetic structure for *P. hupehensis* from K = 2–5 based on the RAD-seq dataset. Ten populations that consistently clustered together were LJL, TSG, QDZ, HKC, HXC, FLC, XJZ, SNJ, SHJZ, and JSZ, which are circled with a dotted line.

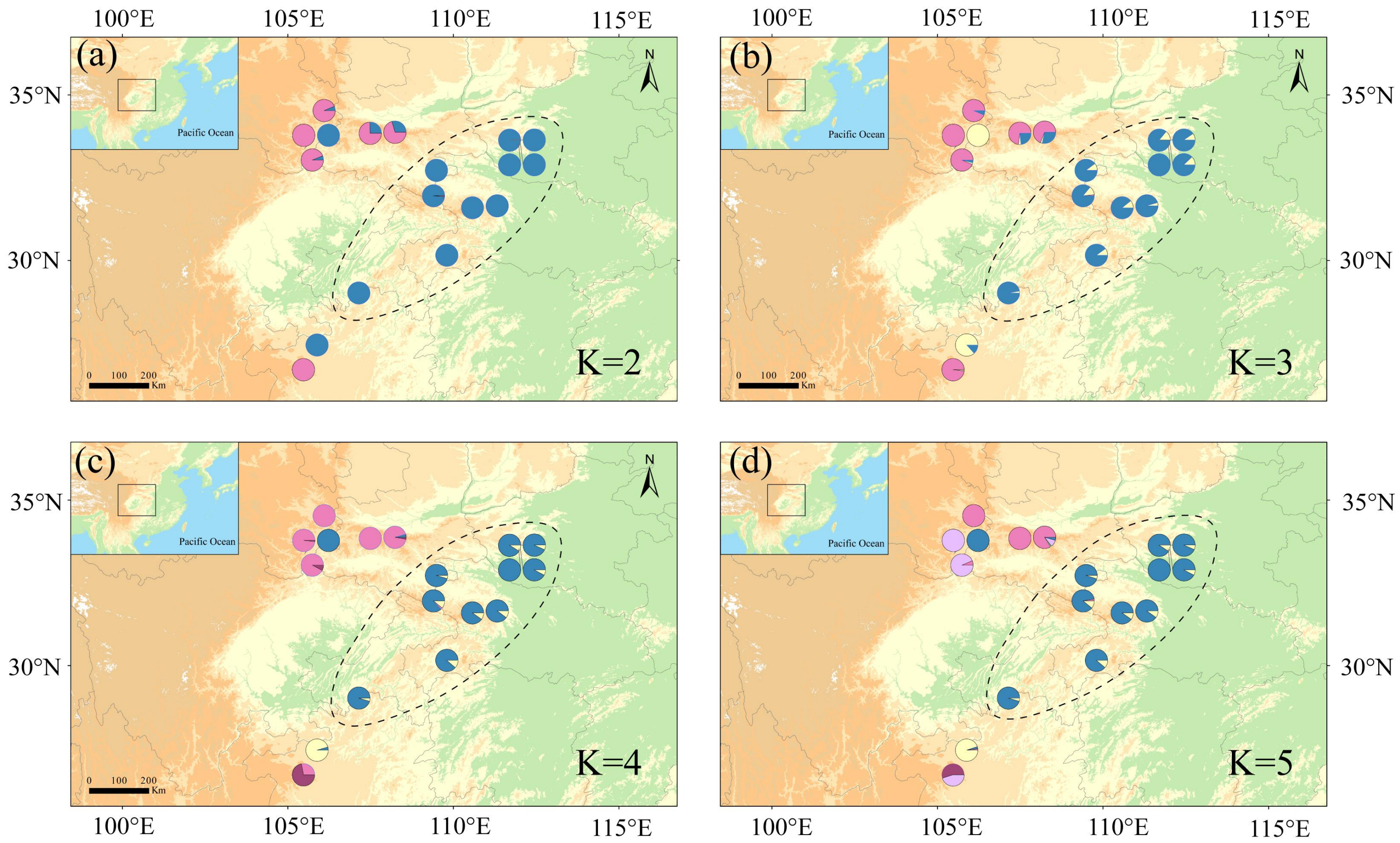

Supplement: Supplementary file 1 — Supplementary data to this article can be found online. [file forres-0024-0005-S1.zip › 10.48130_forres-0024-0005-Suppl-FigureS4.pdf]
