# Supplementary material for: Phylogeography of Pterocarya hupehensis reveals the evolutionary patterns of a Cenozoic relict tree around the Sichuan Basin
Source: For Res (Fayettev). 2024 Mar 12;4:e008. doi: 10.48130/forres-0024-0005 (PMC11524273; doi:10.48130/forres-0024-0005)

Fig. S5 Maximum likelihood tree constructed using 2427 SNPs contained three outgroups (green lines): *Pterocarya macroptera*, *Cyclocarya paliurus*, and *Juglans mandshurica*. The two major lineages of *P. hupehensis* are denoted with pink and blue lines.

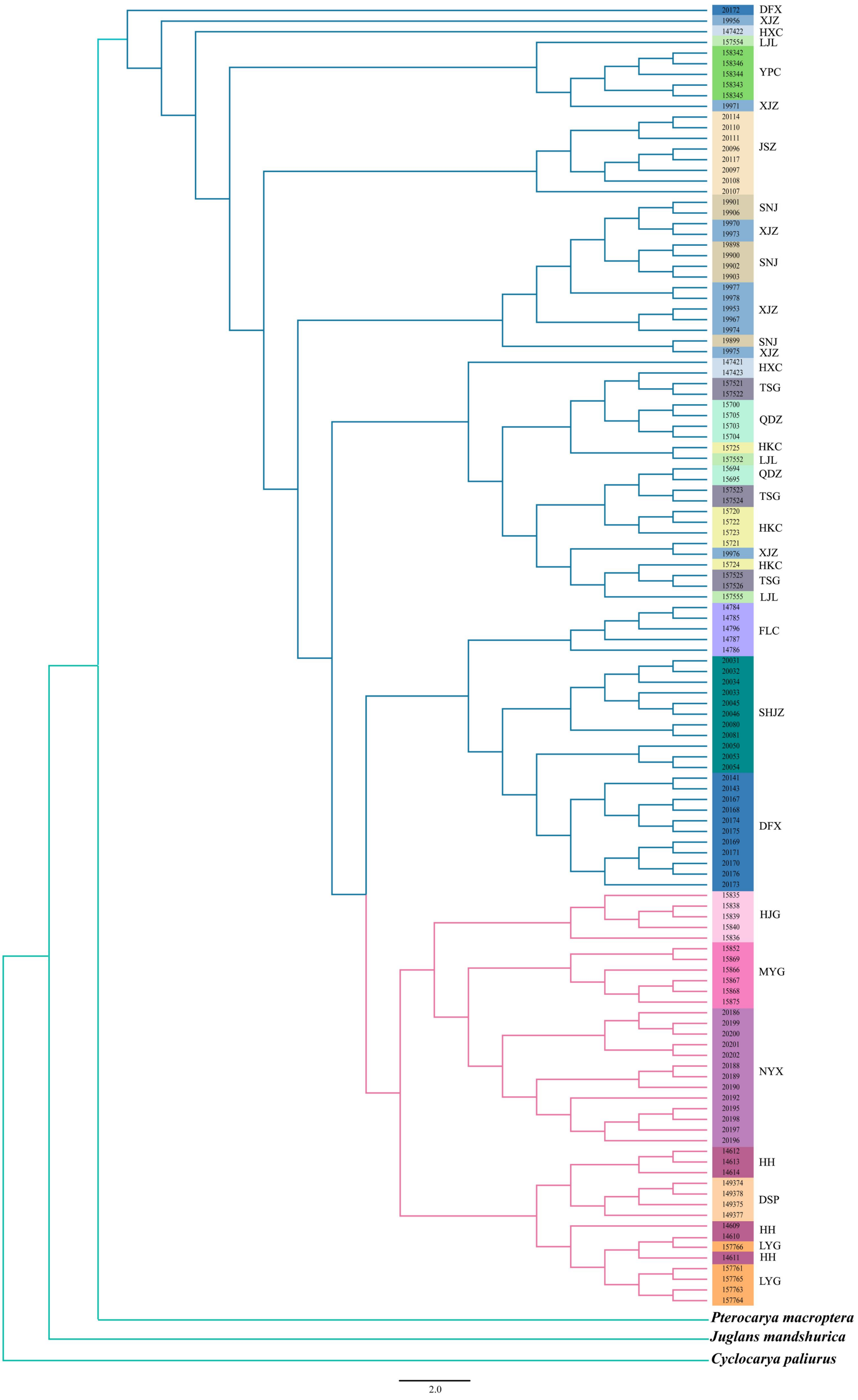

Supplement: Supplementary file 1 — Supplementary data to this article can be found online. [file forres-0024-0005-S1.zip › 10.48130_forres-0024-0005-Suppl-FigureS5.pdf]
