# Supplementary material for: Phylogeography of Pterocarya hupehensis reveals the evolutionary patterns of a Cenozoic relict tree around the Sichuan Basin
Source: For Res (Fayettev). 2024 Mar 12;4:e008. doi: 10.48130/forres-0024-0005 (PMC11524273; doi:10.48130/forres-0024-0005)

**Fig. S7** Demographic history of the western and eastern lineages of *P. hupehensis* inferred by Stairway Plot 2 using unfolded site frequency spectra. The 95% confidence interval for the estimated effective population size is shown by dark gray lines.

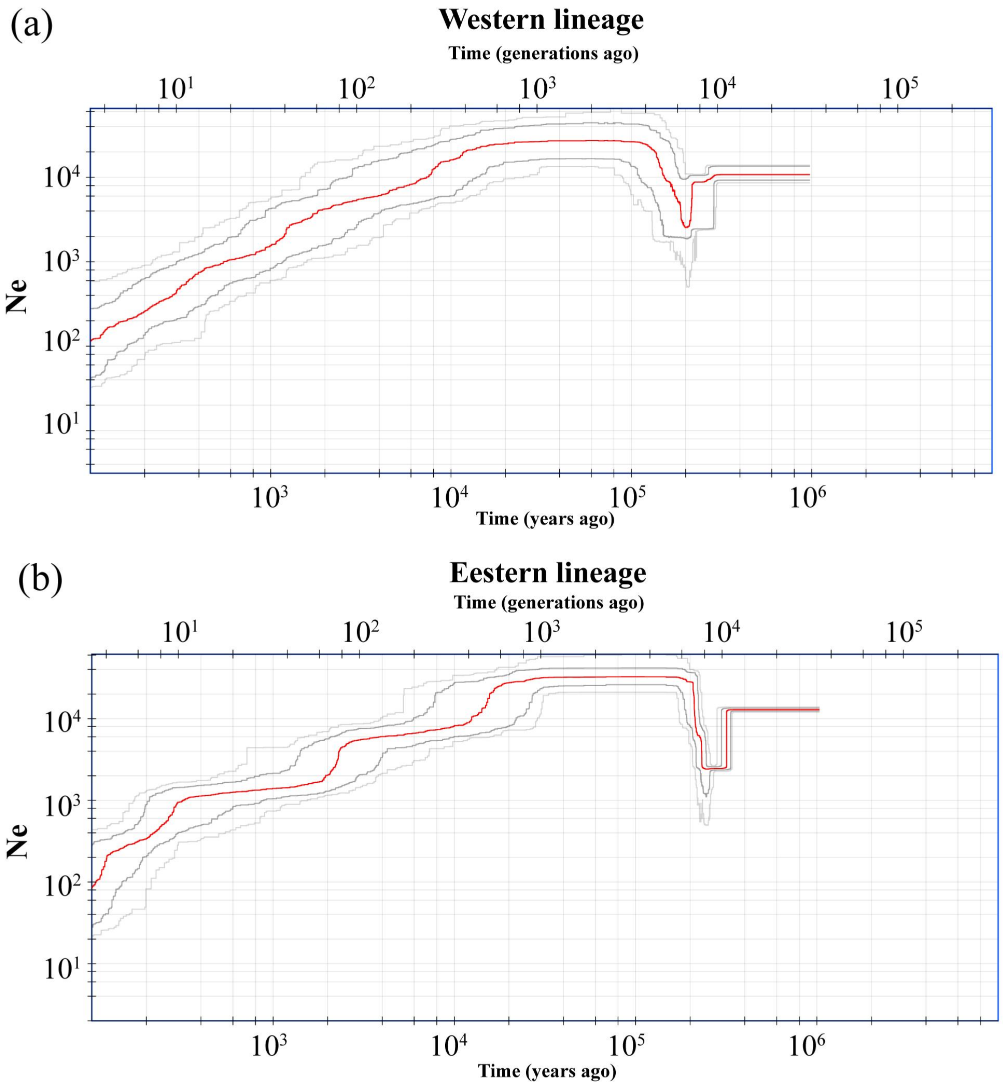

Supplement: Supplementary file 1 — Supplementary data to this article can be found online. [file forres-0024-0005-S1.zip › 10.48130_forres-0024-0005-Suppl-FigureS7.pdf]
