# Supplementary material for: Phylogeography of Pterocarya hupehensis reveals the evolutionary patterns of a Cenozoic relict tree around the Sichuan Basin
Source: For Res (Fayettev). 2024 Mar 12;4:e008. doi: 10.48130/forres-0024-0005 (PMC11524273; doi:10.48130/forres-0024-0005)

Fig. S8 Area Under Curve (AUC) values of Receiver Operating Characteristic (ROC) curves for four time periods. (a) Present climate (AUC = 0.097). (b) LGM period (AUC = 0.978). (c) LIG period (AUC = 0.981). (d) Future 2070 (2061–2080) RCP 4.5 (AUC = 0.982). The higher the value, the better the accuracy of the model.

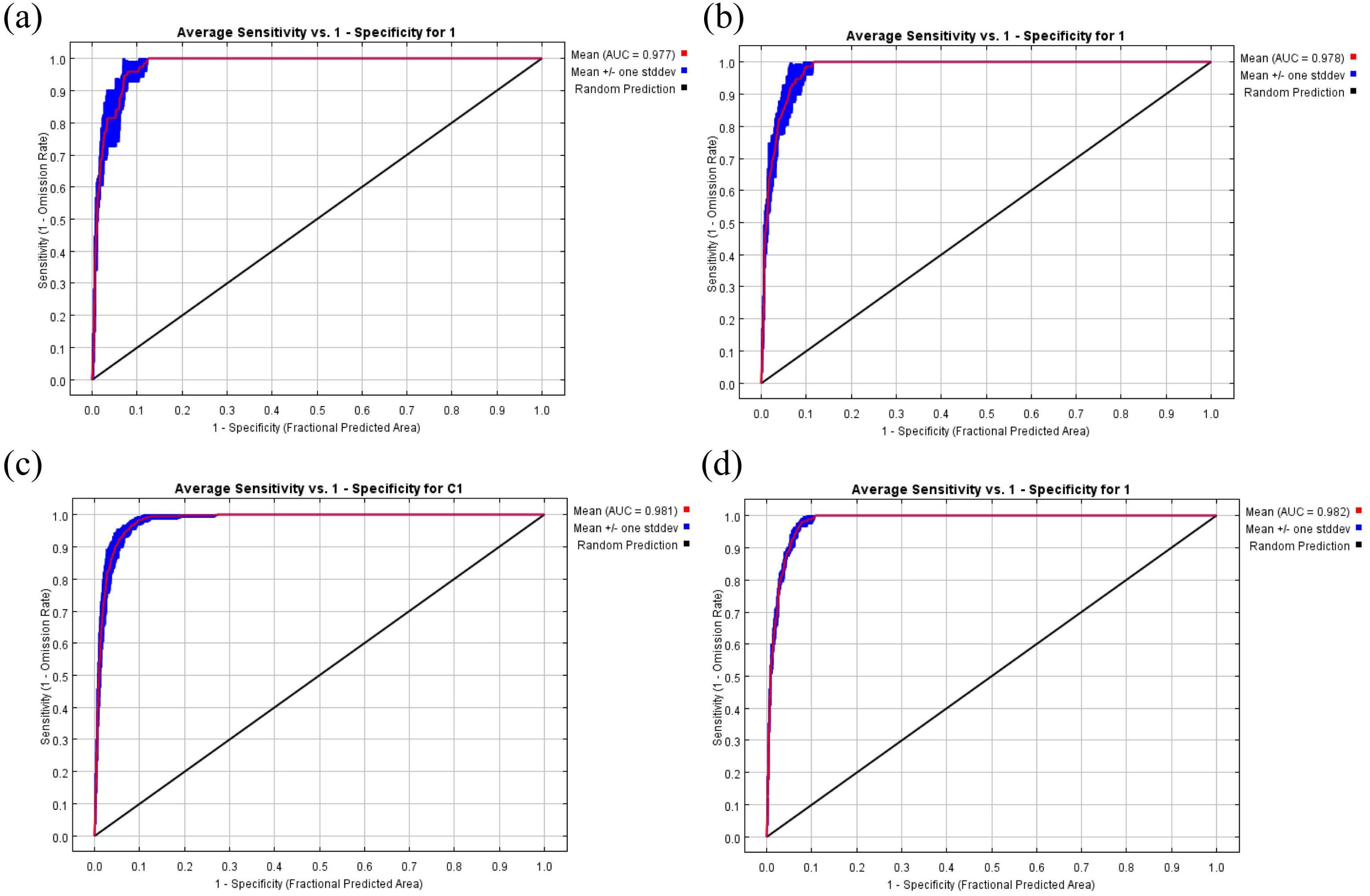

Supplement: Supplementary file 1 — Supplementary data to this article can be found online. [file forres-0024-0005-S1.zip › 10.48130_forres-0024-0005-Suppl-FigureS8.pdf]
