# Supplementary material for: Phylogeography of Pterocarya hupehensis reveals the evolutionary patterns of a Cenozoic relict tree around the Sichuan Basin
Source: For Res (Fayettev). 2024 Mar 12;4:e008. doi: 10.48130/forres-0024-0005 (PMC11524273; doi:10.48130/forres-0024-0005)

Fig. S9 (a) MaxEnt Jackknife tests of the environmental variables importance of *Pterocarya hupehensis* ecological niche model. (b) Marginal response curves of the predicted probability of occurrence for explanatory variables that contributed substantially to the MaxEnt model.

(a)

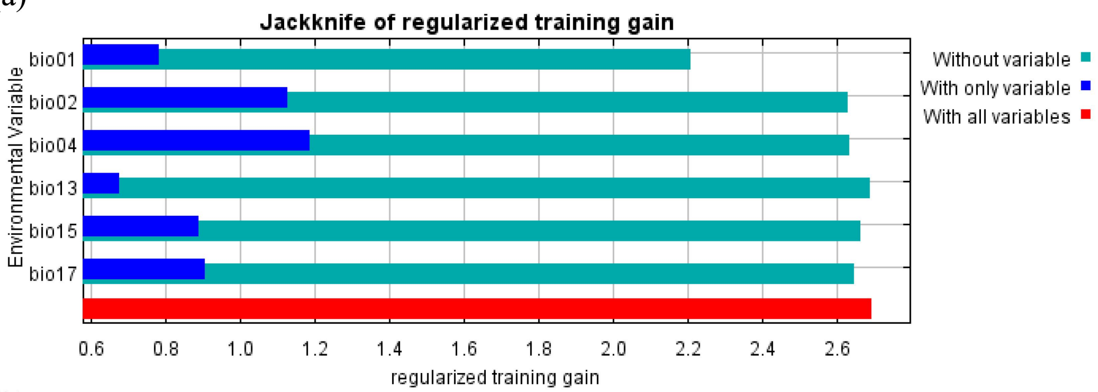

(b)

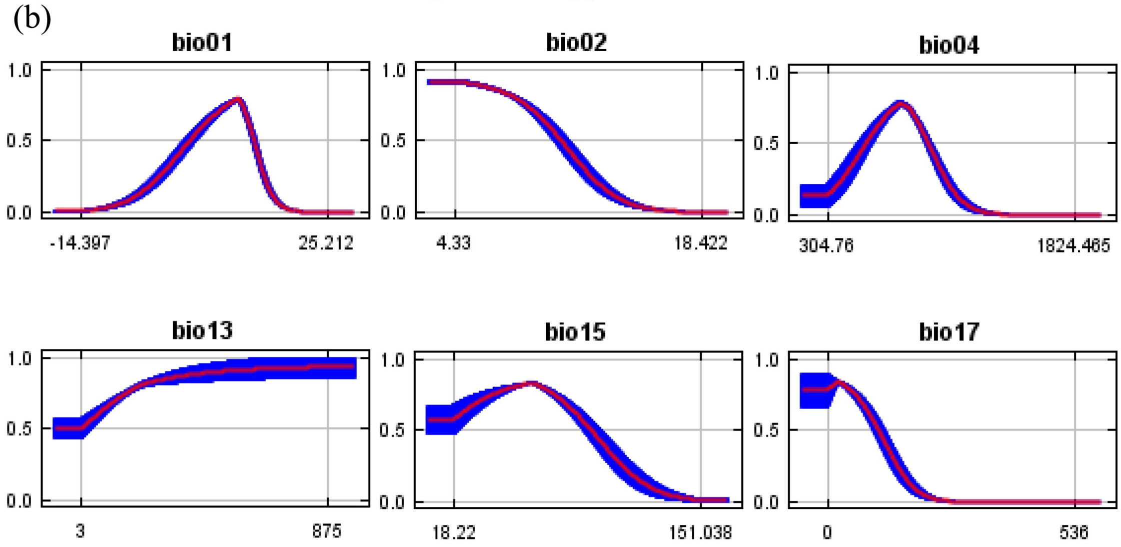

Supplement: Supplementary file 1 — Supplementary data to this article can be found online. [file forres-0024-0005-S1.zip › 10.48130_forres-0024-0005-Suppl-FigureS9.pdf]
