# Supplementary material for: Phylogeography of Pterocarya hupehensis reveals the evolutionary patterns of a Cenozoic relict tree around the Sichuan Basin
Source: For Res (Fayettev). 2024 Mar 12;4:e008. doi: 10.48130/forres-0024-0005 (PMC11524273; doi:10.48130/forres-0024-0005)
Supplement: Supplementary file 1 — Supplementary data to this article can be found online. [file forres-0024-0005-S1.zip › 10.48130_forres-0024-0005-Suppl-TableS1.docx]

**Table S1** Individuals and populations used in this study.

| Population | Chloroplast (individual ID) | RAD (individual ID) |
| --- | --- | --- |
| HH | 14609 | 14609 |
| HH | 14610 | 14610 |
| HH | 14611 | 14611 |
| HH | 14612 | 14612 |
| HH | 14613 | 14613 |
| HH | 14614 | 14614 |
| HH | 14615 | —— |
| HH | 14616 | —— |
| HH | 14617 | —— |
| HH | 14618 | —— |
| HXC | 147421 | 147421 |
| HXC | 147422 | 147422 |
| HXC | 147423 | 147423 |
| HXC | 147424 | —— |
| HXC | 147425 | —— |
| HXC | 147427 | —— |
| HXC | 147428 | —— |
| HXC | 147429 | —— |
| HXC | 1474210 | —— |
| FLC | 14784 | 14784 |
| FLC | 14785 | 14785 |
| FLC | 14786 | 14786 |
| FLC | 14787 | 14787 |
| FLC | 14788 | 14796 |
| FLC | 14790 | —— |
| FLC | 14791 | —— |
| FLC | 14793 | —— |
| FLC | 14796 | —— |
| DSP | 149371 |  |
| DSP | 149372 |  |
| DSP | 149373 |  |
| DSP | 149374 | 149374 |
| DSP | 149375 | 149375 |
| DSP | 149376 | 149377 |
| DSP | 149377 | 149378 |
| DSP | 149378 |  |
| QDZ | 15694 | 15694 |
| QDZ | 15695 | 15695 |
| QDZ | 15700 | 15700 |
| QDZ | 15703 | 15703 |
| QDZ | 15704 | 15704 |
| QDZ | 15705 | 15705 |
| QDZ | 15716 |  |
| QDZ | 15717 |  |
| QDZ | 15718 |  |
| HKC | 15720 | 15720 |
| HKC | 15721 | 15721 |
| HKC | 15722 | 15722 |
| HKC | 15723 | 15723 |
| HKC | 15724 | 15724 |
| HKC | 15725 | 15725 |
| HKC | 15726 |  |
| HKC | 15727 |  |
| HKC | 15730 |  |
| TSG | 157521 | 157521 |
| TSG | 157522 | 157522 |
| TSG | 157523 | 157523 |
| TSG | 157524 | 157524 |
| TSG | 157525 | 157525 |
| TSG | 157526 | 157526 |
| LJL | 157551 | —— |
| LJL | 157552 | 157552 |
| LJL | 157554 | 157554 |
| LJL | 157555 | 157555 |
| LYG | 157761 | 157761 |
| LYG | 157763 | 157763 |
| LYG | 157764 | 157764 |
| LYG | 157765 | 157765 |
| LYG | 157766 | 157766 |
| LYG | 15800 | —— |
| LYG | 15801 | —— |
| LYG | 15802 | —— |
| LYG | 15803 | —— |
| LYG | 15804 | —— |
| YPC | 158342 | 158342 |
| YPC | 158343 | 158343 |
| YPC | 158344 | 158344 |
| YPC | 158345 | 158345 |
| YPC | 158346 | 158346 |
| YPC | 158347 | —— |
| YPC | 158348 | —— |
| HJG | 15835 | 15835 |
| HJG | 15836 | 15836 |
| HJG | 15837 | —— |
| HJG | 15838 | 15838 |
| HJG | 15839 | 15839 |
| HJG | 15840 | 15840 |
| HJG | 15841 | —— |
| MYG | 15852 | 15852 |
| MYG | 15866 | 15866 |
| MYG | 15867 | 15867 |
| MYG | 15868 | 15868 |
| MYG | 15869 | 15869 |
| MYG | 15875 | 15875 |
| SNJ | 19898 | 19898 |
| SNJ | 19899 | 19899 |
| SNJ | 19900 | 19900 |
| SNJ | 19901 | 19901 |
| SNJ | 19902 | 19902 |
| SNJ | 19903 | 19903 |
| SNJ | 19906 | 19906 |
| XJZ | 19953 | 19953 |
| XJZ | —— | 19956 |
| XJZ | 19967 | 19967 |
| XJZ | 19970 | 19970 |
| XJZ | 19971 | 19971 |
| XJZ | 19973 | 19973 |
| XJZ | 19974 | 19974 |
| XJZ | 19975 | 19975 |
| XJZ | 19976 | 19976 |
| XJZ | 19977 | 19977 |
| XJZ | 19978 | 19978 |
| SHJZ | 20031 | 20031 |
| SHJZ | 20032 | 20032 |
| SHJZ | 20033 | 20033 |
| SHJZ | 20034 | 20034 |
| SHJZ | 20045 | 20045 |
| SHJZ | —— | 20046 |
| SHJZ | 20050 | 20050 |
| SHJZ | 20053 | 20053 |
| SHJZ | 20054 | 20054 |
| SHJZ | 20080 | 20080 |
| SHJZ | 20081 | 20081 |
| JSZ | —— | 20114 |
| JSZ | —— | 20096 |
| JSZ | —— | 20097 |
| JSZ | —— | 20107 |
| JSZ | —— | 20108 |
| JSZ | —— | 20110 |
| JSZ | —— | 20111 |
| JSZ | —— | 20117 |
| DFX | —— | 20141 |
| DFX | 20143 | 20143 |
| DFX | 20167 | 20167 |
| DFX | 20168 | 20168 |
| DFX | 20169 | 20169 |
| DFX | 20170 | 20170 |
| DFX | 20171 | 20171 |
| DFX | 20172 | 20172 |
| DFX | —— | 20173 |
| DFX | 20174 | 20174 |
| DFX | 20175 | 20175 |
| DFX | 20176 | 20176 |
| NYX | 20186 | 20186 |
| NYX | 20188 | 20188 |
| NYX | 20189 | 20189 |
| NYX | 20190 | 20190 |
| NYX | 20192 | 20192 |
| NYX | 20195 | 20195 |
| NYX | —— | 20196 |
| NYX | —— | 20197 |
| NYX | —— | 20198 |
| NYX | 20199 | 20199 |
| NYX | 20200 | 20200 |
| NYX | 20201 | 20201 |
| NYX | 20202 | 20202 |
