# Supplementary material for: Phylogeography of Pterocarya hupehensis reveals the evolutionary patterns of a Cenozoic relict tree around the Sichuan Basin
Source: For Res (Fayettev). 2024 Mar 12;4:e008. doi: 10.48130/forres-0024-0005 (PMC11524273; doi:10.48130/forres-0024-0005)
Supplement: Supplementary file 1 — Supplementary data to this article can be found online. [file forres-0024-0005-S1.zip › 10.48130_forres-0024-0005-Suppl-TableS2.docx]

**Table S2** Mapping rates to the reference genome and read numbers for individual samples.

| **Individual ID** | **Mapping rate** | **Number of reads** |
| --- | --- | --- |
| 14609 | 86.33% | 6552296 |
| 14610 | 53.11% | 5172034 |
| 14611 | 62.88% | 4073139 |
| 14612 | 69.21% | 3800102 |
| 14613 | 63.74% | 2472061 |
| 14614 | 93.15% | 2342204 |
| 147421 | 94.58% | 2175566 |
| 147422 | 91.18% | 2093201 |
| 147423 | 49.44% | 1694543 |
| 14784 | 64.04% | 1168946 |
| 14785 | 68.97% | 1244608 |
| 14786 | 80.48% | 1126562 |
| 14787 | 68.15% | 990717 |
| 14796 | 83.61% | 1183543 |
| 149374 | 78.32% | 1218511 |
| 149375 | 71.98% | 1185754 |
| 149377 | 73.6% | 995503 |
| 149378 | 67.13% | 773199 |
| 15694 | 92.67% | 1121764 |
| 15695 | 88.69% | 1085859 |
| 15700 | 78.64% | 1193089 |
| 15703 | 88.31% | 1156744 |
| 15704 | 89.43% | 1097480 |
| 15705 | 87.7% | 1118011 |
| 15720 | 90.33% | 1151781 |
| 15721 | 86.12% | 766578 |
| 15722 | 93.17% | 673748 |
| 15723 | 91.02% | 768910 |
| 15724 | 86.42% | 883989 |
| 15725 | 91.76% | 700852 |
| 157521 | 90.94% | 651887 |
| 157522 | 67.74% | 855977 |
| 157523 | 72.43% | 745972 |
| 157524 | 89.23% | 715532 |
| 157525 | 88.18% | 660379 |
| 157526 | 86.11% | 414639 |
| 157552 | 80.64% | 722778 |
| 157554 | 89.34% | 451484 |
| 157555 | 84.47% | 722970 |
| 157761 | 92.8% | 699780 |
| 157763 | 91.96% | 494203 |
| 157764 | 86.2% | 744498 |
| 157765 | 82.63% | 490153 |
| 157766 | 61.39% | 873274 |
| 158342 | 88.48% | 637230 |
| 158343 | 89.9% | 496157 |
| 158344 | 90.45% | 650718 |
| 158345 | 86.85% | 592034 |
| 158346 | 85.48% | 729593 |
| 15835 | 85.47% | 627188 |
| 15836 | 85% | 338966 |
| 15838 | 84.27% | 540482 |
| 15839 | 78.95% | 386396 |
| 15840 | 91.65% | 582432 |
| 15852 | 86.64% | 657601 |
| 15866 | 89.83% | 170228 |
| 15867 | 93.59% | 377730 |
| 15868 | 92.78% | 261018 |
| 15869 | 88.64% | 423990 |
| 15875 | 90.29% | 221869 |
| 19901 | 91.43% | 213513 |
| 19976 | 84.56% | 237993 |
| 20114 | 75.97% | 226918 |
| 19898 | 94.26% | 458930 |
| 19899 | 93.18% | 466386 |
| 19900 | 94.39% | 330308 |
| 19902 | 95.44% | 357331 |
| 19903 | 94.66% | 445207 |
| 19906 | 90.65% | 399380 |
| 19953 | 91.19% | 402035 |
| 19956 | 89.88% | 285741 |
| 19967 | 94.81% | 350500 |
| 19970 | 93.75% | 321960 |
| 19971 | 92.38% | 373560 |
| 19973 | 90.5% | 392835 |
| 19974 | 92.75% | 352613 |
| 19975 | 93.05% | 452999 |
| 19977 | 92.03% | 347936 |
| 19978 | 92.92% | 377519 |
| 20031 | 95.25% | 401679 |
| 20032 | 89.3% | 251733 |
| 20033 | 95.47% | 391820 |
| 20034 | 96.2% | 237024 |
| 20045 | 85.91% | 315325 |
| 20046 | 92.58% | 312720 |
| 20050 | 85.64% | 284324 |
| 20053 | 92.1% | 429860 |
| 20054 | 89.58% | 364797 |
| 20080 | 85.07% | 365087 |
| 20081 | 80.33% | 165163 |
| 20096 | 84.42% | 391810 |
| 20097 | 81.4% | 353139 |
| 20107 | 81.02% | 131791 |
| 20108 | 75.08% | 263126 |
| 20110 | 90.41% | 335328 |
| 20111 | 90.25% | 452581 |
| 20117 | 90.58% | 258204 |
| 20141 | 90.99% | 285031 |
| 20143 | 89.28% | 233491 |
| 20167 | 89.98% | 229954 |
| 20168 | 94.18% | 285315 |
| 20169 | 93.73% | 307231 |
| 20170 | 91.48% | 294846 |
| 20171 | 93.91% | 268159 |
| 20172 | 94.17% | 238984 |
| 20173 | 90% | 218371 |
| 20174 | 88.6% | 319447 |
| 20175 | 89.6% | 267206 |
| 20176 | 94.09% | 278924 |
| 20186 | 92.7% | 245451 |
| 20188 | 94.45% | 265044 |
| 20189 | 93.77% | 151440 |
| 20190 | 92.59% | 145731 |
| 20192 | 95.07% | 139687 |
| 20195 | 98.23% | 216136 |
| 20196 | 95.08% | 336611 |
| 20197 | 95.47% | 308028 |
| 20198 | 95.74% | 139262 |
| 20199 | 93.86% | 174731 |
| 20200 | 93.83% | 309870 |
| 20201 | 95.57% | 260485 |
| 20202 | 95.78% | 135137 |
| 14676 | 69.64% | 142605 |
| 16462 | 80.73% | 159324 |
| CS01624 | 82.61% | 165426 |
| **Mean** | **86.72008%** | **703516.432** |
| **Max** | **98.23%** | **6552296** |
| **Minimum** | **49.44%** | **131791** |
