# Supplementary material for: Phylogeography of Pterocarya hupehensis reveals the evolutionary patterns of a Cenozoic relict tree around the Sichuan Basin
Source: For Res (Fayettev). 2024 Mar 12;4:e008. doi: 10.48130/forres-0024-0005 (PMC11524273; doi:10.48130/forres-0024-0005)
Supplement: Supplementary file 1 — Supplementary data to this article can be found online. [file forres-0024-0005-S1.zip › 10.48130_forres-0024-0005-Suppl-TableS3.docx]

**Table S3** Analysis of migration events from 1 to 10 performed with TreeMix software. The standard error represents the standard error for all entries in the covariance matrix estimated from the data. The variance explained represents the proportion of the variance in relatedness between populations explained by the model.

| **Number of migration events** | **Standard error** | **Variance explained** |
| --- | --- | --- |
| 1 | 5.965E−05 | 0.889 |
| 2 | 5.965E−05 | 0.916 |
| 3 | 5.965E−05 | 0.9176 |
| 4 | 5.965E−05 | 0.929 |
| 5 | 5.965E−05 | 0.934 |
| 6 | 5.965E−05 | 0.938 |
| 7 | 5.965E−05 | 0.945 |
| 8 | 5.965E−05 | 0.940 |
| 9 | 5.965E−05 | 0.945 |
| 10 | 5.965E−05 | 0.950 |
