# Supplementary material for: Phylogeography of Pterocarya hupehensis reveals the evolutionary patterns of a Cenozoic relict tree around the Sichuan Basin
Source: For Res (Fayettev). 2024 Mar 12;4:e008. doi: 10.48130/forres-0024-0005 (PMC11524273; doi:10.48130/forres-0024-0005)
Supplement: Supplementary file 1 — Supplementary data to this article can be found online. [file forres-0024-0005-S1.zip › 10.48130_forres-0024-0005-Suppl-TableS4.docx]

**Table S4** Relative contributions of environmental variables to the MaxEnt model.

| Bioclimatic variable | Variable code | Percent contribution | Permutation importance |
| --- | --- | --- | --- |
| Annual Mean Temperature | BIO1 | 35% | 17 |
| Mean Diurnal Range [Mean of monthly (max temp−min temp)] | BIO2 | 26% | 19.1 |
| Precipitation of Driest Quarter | BIO17 | 16.1% | 15.1 |
| Temperature Seasonality (standard deviation × 100) | BIO4 | 14.3% | 40.6 |
| Precipitation Seasonality (Coefficient of Variation) | BIO15 | 8.5% | 6.8 |
| Precipitation of Wettest Month | BIO13 | 0.2% | 1.5 |
